# Novel Aurone Derivative Ameliorates MASH Lipid Metabolism via the AMPK-ACC-PPARα Axis

**DOI:** 10.3390/ijms262211099

**Published:** 2025-11-17

**Authors:** Sule Bai, Yi Zou, Wenyi Zhang, Jiajia Yu, Zhenzhen Qie, Zhen Liu, Peng Yu, Cen Xiang, Yuou Teng

**Affiliations:** School of Biological Engineering, Tianjin University of Science and Technology, Tianjin 300457, Chinayupeng@tust.edu.cn (P.Y.)

**Keywords:** MASH, lipid metabolism, inflammation, AMPK-ACC-PPARα axis

## Abstract

Metabolic dysfunction-associated steatohepatitis (MASH), a progressive form of metabolic dysfunction-associated fatty liver disease (MASLD), is characterized by disrupted lipid metabolism and persistent inflammation, which can lead to cirrhosis and hepatocellular carcinoma. The novel pan-peroxisome proliferator-activated receptor (PPAR) agonist **1d** has been previously shown to alleviate insulin resistance and hepatic steatosis in type 2 diabetic (T2DM) mice; however, its mechanism of action remains unclear. Our integrated in vitro and in vivo findings demonstrate that compound **1d** significantly improves disordered hepatic lipid metabolism by modulating the AMPK-ACC-PPARα axis—specifically, by upregulating AMPK expression and phosphorylation, inhibiting ACC activity, downregulating FASN, and upregulating PPARα. Concurrently, **1d** exhibits potent anti-inflammatory effects in both settings, effectively mitigating hepatic inflammation in a MASH mouse model. Therefore, compound **1d** improves lipid metabolism through the AMPK-ACC-PPARα axis and additionally provides an anti-inflammatory benefit, highlighting its potential as a novel therapeutic candidate for MASH.

## 1. Introduction

Metabolic dysfunction-associated steatotic liver disease (MASLD), a prevalent hepatic condition affecting over one-third of adults worldwide, is primarily driven by unhealthy lifestyle choices, dyslipidemia, and metabolic syndrome [1,2,3]. The disease spectrum ranges from simple fatty liver (SFL) to metabolic dysfunction-associated steatohepatitis (MASH), and may further progress to cirrhosis, hepatic failure, and hepatocellular carcinoma (HCC) [4,5,6]. The initial stage is characterized by hepatic lipid accumulation, predominantly in the form of triglyceride (TG) deposition, which marks both the onset of the disease and a major driver of its advancement [5,7]. Without timely intervention, persistent lipid overload can progress to MASH. Notably, 40–50% of MASH patients develop liver fibrosis—the primary predictor of mortality in this population [8,9]. Although the direct oncogenic potential of isolated hepatic steatosis requires further validation, MASH is unequivocally established as a significant risk factor for HCC, even in the absence of detectable fibrosis [10,11,12]. The disease trajectory often becomes irreversible upon progression to cirrhosis or HCC, typically leaving liver transplantation as the only viable treatment. The widely accepted “multiple-hit hypothesis” explains MASLD pathogenesis as a synergistic process involving insulin resistance, hepatic lipid accumulation, chronic inflammation, and oxidative stress [13,14,15]. This intricate pathogenic network underlies the considerable challenges in developing effective therapies.

**Figure 1:**
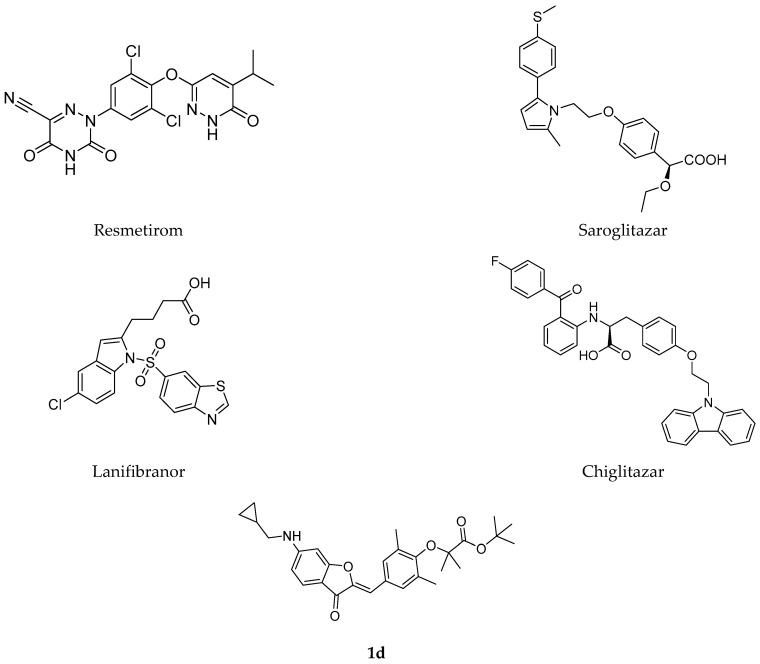
Chemical structures of the relevant compounds.

Currently, the thyroid hormone receptor-β (THR-β) agonist Resmetirom (MGL-3196) remains the only FDA-approved pharmacotherapy for non-cirrhotic adults with moderate-to-severe fibrotic MASH (Figure 1) [16]. Resmetirom, a selective THR-β agonist, demonstrates multi-faceted efficacy by reducing hepatic fat, triglycerides, and key atherogenic lipids (LDL-C, ApoB, ApoC-III), while improving serum transaminases and histopathological markers. Its hepatic benefits occur without THR-α-related cardiac or skeletal effects [17,18,19,20], though transient gastrointestinal side effects warrant further safety evaluation [18,19].

Peroxisome proliferator-activated receptors (PPARs) are ligand-dependent transcription factors comprising three subtypes—PPARα, PPARδ (also known as PPARβ), and PPARγ [21,22]. Among them, PPARα is highly expressed in metabolic organs such as the liver, heart, kidneys, brown adipose tissue, and skeletal muscle. It plays a central role in energy homeostasis by regulating genes involved in fatty acid β-oxidation, microsomal ω-oxidation, and lipid transport [23,24,25,26,27]. Given its pivotal functions in energy metabolism and inflammatory regulation, the PPAR signaling pathway has emerged as a promising therapeutic target for MASH [28,29,30,31]. Reflecting this potential, several PPAR-targeting agents—including Saroglitazar, Lanifibranor, and Chiglitazar (Figure 1)—have advanced to phase III clinical trials for MASH, highlighting the growing translational relevance of PPAR modulation in this field [32,33,34].

In recent years, AMP-activated protein kinase (AMPK) has gained recognition as a promising therapeutic target for metabolic disorders such as diabetes, obesity, and fatty liver disease, due to its central role in regulating cellular energy homeostasis [35,36,37,38]. AMPK directly phosphorylates acetyl-CoA carboxylase (ACC) at Ser79, leading to the suppression of its enzymatic activity [39]. As the rate-limiting enzyme that catalyzes the conversion of acetyl-CoA to malonyl-CoA—the initial and committed step in fatty acid biosynthesis—ACC inhibition subsequently downregulates fatty acid synthase (FASN) expression, thereby reducing de novo lipogenesis. Notably, this FASN downregulation is more frequently observed in metabolic pathways associated with lipid metabolism improvement, in contrast to FASN inhibitors primarily used for anticancer purposes [40,41,42]. Concurrently, this signaling cascade upregulates carnitine palmitoyltransferase 1 (CPT1), a key mediator of mitochondrial fatty acid β-oxidation, thereby promoting fatty acid catabolism [35,43]. Moreover, studies have shown that AMPK activation enhances PPARα expression, which further stimulates fatty acid oxidation [44]. Based on this mechanistic framework, targeting the AMPK-ACC-PPARα axis to ameliorate hepatic lipid metabolic dysregulation has emerged as a key therapeutic strategy for MASH [43,45].

In our previous study, we developed a novel pan-PPAR agonist compound **1d** via a functional group-oriented design strategy (Figure 1). This agent significantly improved insulin resistance in HFD/STZ-induced mice with type 2 diabetes mellitus (T2DM), while completely avoiding two hallmark adverse effects associated with Thiazolidinediones—weight gain and fluid retention. It also concurrently improved lipid metabolism and attenuated hepatic steatosis [46]. In the present work, we further demonstrate that **1d** alleviates MASH pathology in both cellular and animal models by modulating the AMPK-ACC-PPARα axis, thus offering not only a promising therapeutic candidate but also a mechanistic rationale for MASH treatment.

## 2. Result

### 2.1. ***1d*** Ameliorates FFAs-Induced Lipid Accumulation in L02 Hepatocytes

**Figure 2:**
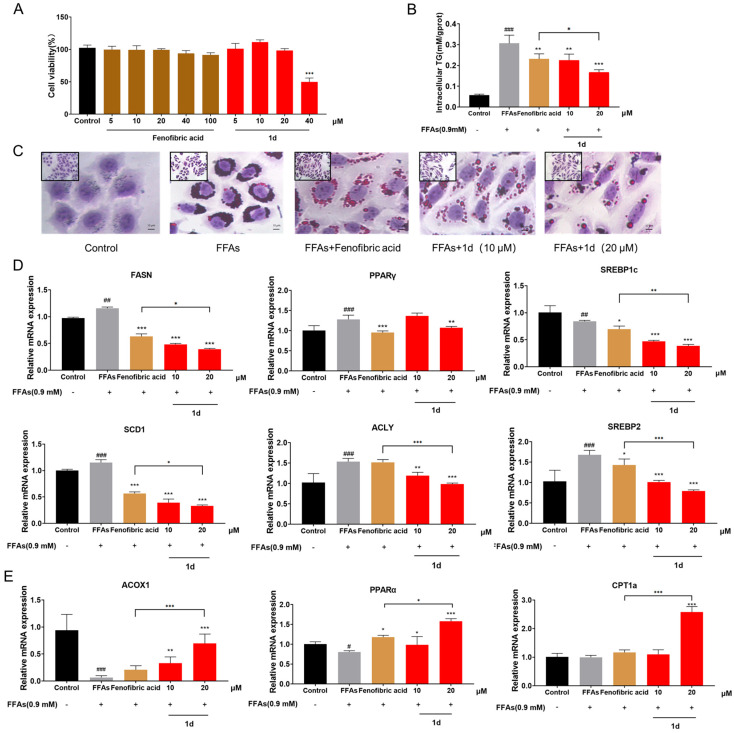
**1d** significantly ameliorates lipid accumulation in the FFAs-induced L02 hepatocyte steatosis model. (**A**) Cell viability of L02 cells after 24 h compound treatment. (**B**) Intracellular triglyceride (TG) levels. (**C**) Oil Red O staining of lipid droplets (400×, scale bar = 50 μm). (**D**) mRNA levels of fatty acid synthesis-related genes. (**E**) mRNA levels of β-oxidation-related genes. Data are presented as mean ± SD (*n* = 3). Statistical significance: ^#^ *p* < 0.05, ^##^ *p* < 0.01, ^###^ *p* < 0.001 vs. Control; * *p* < 0.05, ** *p* < 0.01, *** *p* < 0.001 vs. FFAs. In the bar chart, the colors black, gray, yellow, and red represent the Control group, Model group, Fenofibric acid treatment group, and compound **1d** treatment group, respectively. The color coding is applied to facilitate easier reading, and the groups are also clearly labeled on the x-axis. The symbols “-” and “+” indicate the absence and presence of FFAs treatment.

To determine appropriate compound concentrations, the effect of **1d** on L02 cell viability was first assessed after 24 h exposure. Treatment with up to 20 μM of **1d** showed no significant cytotoxicity (Figure 2A), and this concentration was therefore selected as the maximum dose for subsequent experiments. Intracellular triglyceride (TG) levels were used as a key indicator of lipid accumulation. Exposure to FFAs for 24 h significantly increased TG content in L02 cells (*p* < 0.001). In contrast, treatment with either 100 μM Fenofibric acid or 10–20 μM **1d** significantly attenuated TG accumulation (*p* < 0.01). Notably, 20 μM **1d** exhibited stronger TG-lowering efficacy than 100 μM Fenofibric acid (Figure 2B). Oil Red O staining results corroborated the TG quantification data: while FFAs-treated cells displayed substantial lipid droplet accumulation, both Fenofibric acid and **1d** effectively reduced lipid deposition, with the 20 μM **1d** treatment showing the most pronounced reduction and minimal residual lipid droplets (Figure 2C).

We next examined the mRNA expression of key genes involved in lipid metabolism in L02 cells. Treatment with compound **1d** led to significant downregulation of genes associated with fatty acid synthesis, including *FASN*, *SREBP1c*, *PPARγ*, *SCD1*, and *ACLY* (all *p* < 0.01). Notably, compared to Fenofibric acid, 20 μM **1d** exerted stronger inhibitory effects on all these genes except *PPARγ* (*p* < 0.05; Figure 2D). Furthermore, treatment with 20 μM **1d** markedly upregulated the expression of β-oxidation-related genes *ACOX1*, *PPARα*, and *CPT1a* (*p* < 0.001; Figure 2E).

### 2.2. ***1d*** Ameliorates Mitochondrial Dysfunction Induced by FFAs in L02 Cells

**Figure 3:**
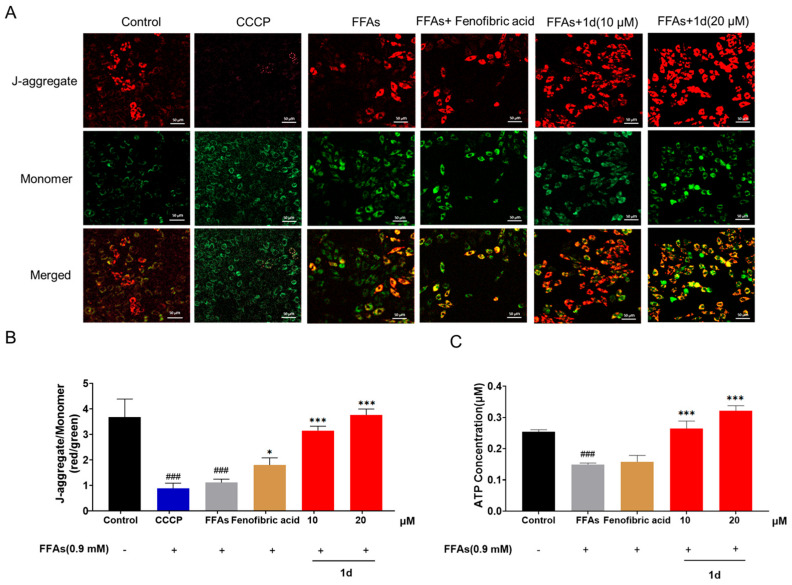
**1d** ameliorates FFAs-induced mitochondrial dysfunction in L02 cells. (**A**) Fluorescence microscopy images showing changes in mitochondrial membrane potential in high-fat-treated L02 cells after compound treatment (400×, scale bar = 50 μm). (**B**) Quantitative analysis of mitochondrial membrane potential based on the ratio of JC-1 aggregate (red) to monomer (green) fluorescence intensity. (**C**) Cellular ATP levels. Data are presented as mean ± SD (*n* = 3). Statistical significance: ^###^ *p* < 0.001 vs. Control; * *p* < 0.05, *** *p* < 0.001 vs. FFAs. In the bar chart, the colors black, gray, yellow, and red represent the Control group, Model group, Fenofibric acid treat-ment group, and compound **1d** treatment group, respectively. The color coding is applied to facilitate easier reading, and the groups are also clearly labeled on the x-axis. The symbols “-” and “+” indicate the absence and presence of FFAs treatment.

Mitochondria, serving as the central hub of cellular energy metabolism, play a pivotal role in the maintenance of lipid homeostasis [47]. Accumulating evidence has demonstrated a close association between mitochondrial dysfunction and the pathogenesis of MASH [48,49,50,51]. Notably, therapeutic strategies aimed at enhancing hepatic mitochondrial function have been shown to significantly alleviate hepatic lipid accumulation and attenuate MASH progression [43,52,53]. To evaluate the impact of compound **1d** on mitochondrial function, this study employed JC-1 fluorescent probe to detect changes in mitochondrial membrane potential (ΔΨm) in a free fatty acids (FFAs)-induced L02 hepatocyte steatosis model. Fluorescence microscopy imaging was further utilized to observe mitochondrial membrane potential dynamics for assessing mitochondrial structural stability, while intracellular ATP levels were measured to comprehensively evaluate mitochondrial functional status. Treatment with CCCP (10 μM) and free fatty acids (FFAs) significantly reduced the JC-1 aggregate (red fluorescence) to monomer (green fluorescence) ratio in L02 cells (*p* < 0.001), indicating severe mitochondrial depolarization and structural instability. In contrast, treatment with compound **1d** (10 and 20 μM) markedly restored the JC-1 aggregate-to-monomer ratio (*p* < 0.001), elevated the mitochondrial membrane potential, and improved mitochondrial stability. Conversely, Fenofibric acid (100 μM) only marginally ameliorated the reduction in mitochondrial membrane potential (*p* < 0.05) (Figure 3A,B). Consistent with these findings, FFAs treatment significantly reduced cellular ATP levels (*p* < 0.001), suggesting mitochondrial dysfunction under lipotoxic conditions. Notably, **1d** treatment effectively restored mitochondrial function and normalized ATP production (*p* < 0.001), while Fenofibric acid showed no significant improvement in ATP levels (Figure 3C).

### 2.3. ***1d*** Attenuates LPS-Induced Inflammatory Response in RAW264.7 Macrophages

**Figure 4:**
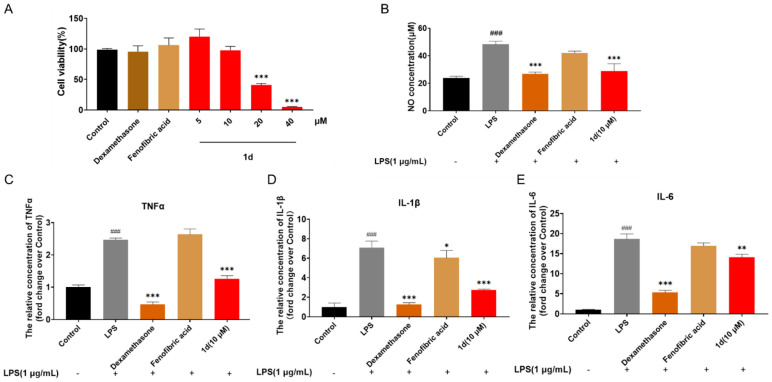
Anti-inflammatory Effects of **1d** in LPS-Stimulated RAW264.7 Macrophages: (**A**) Cell viability following 24-h compound treatment; (**B**) NO levels in culture supernatants of LPS-stimulated RAW264.7 cells after compound treatment; (**C**) TNF-α levels in culture supernatants of LPS-stimulated RAW264.7 cells after compound treatment; (**D**) IL-1β levels in culture supernatants of LPS-stimulated RAW264.7 cells after compound treatment; (**E**) IL-6 levels in culture supernatants of LPS-stimulated RAW264.7 cells after compound treatment. Data are presented as mean ± SD (*n* = 3). Statistical significance: ^###^ *p* < 0.001 vs. Control; * *p* < 0.05, ** *p* < 0.01, *** *p* < 0.001 vs. LPS. The bar chart uses black for the Control group, dark brown for Dexamethasone, light brown for Fenofibric acid, and red for compound **1d**, while “-” and “+” indicate absence or presence of FFAs co-treatment respectively, with all groups explicitly labeled on the x-axis. The figure has been updated to ensure color consistency for the same treat-ment groups across all subplots.

Inflammation plays a pivotal role in the progression of MASH [54]. Lipopolysaccharide (LPS) is widely used to induce robust inflammatory responses in murine RAW264.7 macrophages, representing a classical in vitro model for inflammation studies. Our results showed that 24 h treatment with 10 μM **1d** did not affect cell viability (Figure 4A). Both **1d** and Dexamethasone significantly suppressed the production of the inflammatory mediator nitric oxide (NO) (*p* < 0.001), whereas Fenofibric acid exhibited no inhibitory effect on NO release (Figure 4B). Moreover, **1d** and Dexamethasone similarly and markedly reduced the secretion of pro-inflammatory cytokines TNF-α, IL-1β, and IL-6 (*p* < 0.01). In contrast, Fenofibric acid only slightly attenuated IL-1β levels (*p* < 0.05) (Figure 4C–E).

### 2.4. ***1d*** Ameliorates Lipid Metabolism in MCD Diet-Induced MASH Mice

**Figure 5:**
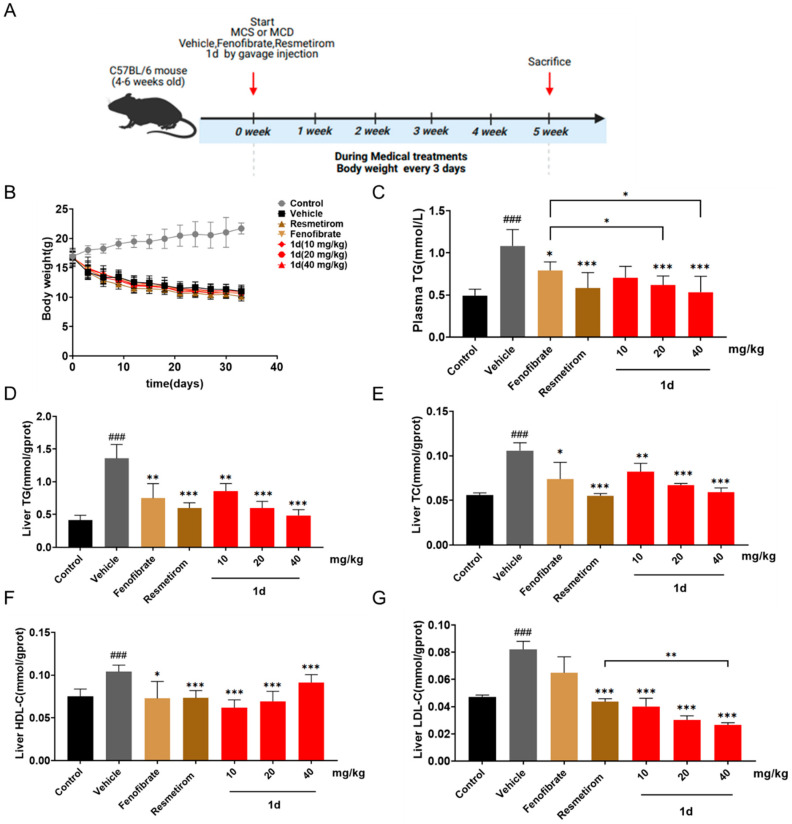
**1d** ameliorates lipid metabolism in MCD diet-induced MASH mice. (**A**) Establishment of MCD diet-induced MASH mouse model and compound intervention. (**B**) Body weight changes during the treatment period. (**C**) Plasma TG levels after treatment. (**D**) Liver TG levels after treatment. (**E**) Liver TC levels after treatment. (**F**) Liver HDLC levels after treatment. (**G**) Liver LDLC levels after treatment. Data are presented as mean ± SD (*n* = 8). Statistical significance: ^###^ *p* < 0.001 vs. Control; * *p* < 0.05, ** *p* < 0.01, *** *p* < 0.001 vs. Vehicle. The color representations are as follows: black denotes the Control group, gray represents the Vehicle group, yellow indicates the Fenofibrate treatment group, brown corresponds to the Resmetirom treatment group, and red signifies the various dosage groups of compound **1d**. All groups are clearly labeled on the X-axis. The color representations are as follows: black denotes the Control group, gray represents the Vehicle group, yellow indicates the Fenofibrate treatment group, brown corresponds to the Resmetirom treatment group, and red signifies the various dosage groups of compound **1d**. All groups are clearly labeled on the X-axis.

Building on the demonstrated efficacy of compound **1d** in reducing lipid accumulation and improving metabolic parameters in vitro, we further evaluated its effects on lipid metabolism in MCD diet-induced MASH mice (Figure 5A). In addition to fenofibrate, Resmetirom—a currently approved MASH therapeutic—was included as a positive control to benchmark the in vivo performance of **1d**. Throughout the treatment period, MCD diet-induced weight loss did not differ significantly between the Vehicle group and any drug-treated group (Figure 5B). All treatment groups exhibited varying degrees of improvement in plasma TG levels. Notably, both the 20 mg/kg and 40 mg/kg doses of **1d** led to a more pronounced reduction in plasma TG relative to the Fenofibrate group (*p* < 0.05), achieving efficacy comparable to that of Resmetirom (Figure 5C). Moreover, liver TG and TC levels were more substantially improved in the **1d**-treated groups than in the Fenofibrate group (*p* < 0.05; Figure 5D,E). Of particular interest, the 40 mg/kg **1d** regimen not only lowered liver LDL-C more markedly than Resmetirom (*p* < 0.01) but also elevated liver HDL-C levels (Figure 5F,G).

### 2.5. ***1d*** Ameliorates Inflammation and Liver Histopathological Features in MCD Diet-Induced MASH Mice

**Figure 6:**
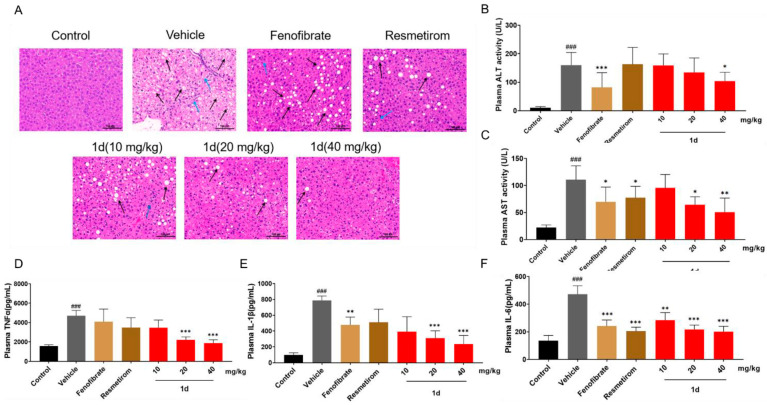
**1d** ameliorates inflammation and liver histopathological features in MCD diet-induced MASH mice. (**A**) Representative H&E-stained liver sections after treatment (200×, scale bar = 100 μm). (**B**) Plasma ALT activity levels after treatment. (**C**) Plasma AST activity levels after treatment. (**D**) Plasma TNFα levels after treatment. (**E**) Plasma IL-1β levels after treatment. (**F**) Plasma IL-6 levels after treatment. Data are presented as mean ± SD (*n* = 8). Statistical significance: ^###^ *p* < 0.001 vs. Control; * *p* < 0.05, ** *p* < 0.01, *** *p* < 0.001 vs. Vehicle. In the H&E staining images, black arrows highlight ballooning degeneration of hepatocytes while blue arrows point to inflammatory infiltration, with both pathological features being explained in the main text. The color scheme and group representations in the bar chart remain consistent with the descriptions provided for Figure 5.

Steatosis and inflammation represent hallmark pathological manifestations in MASH-affected livers. In mouse livers, MCD diet feeding induced characteristic hepatocellular ballooning (indicated by black arrows) and inflammatory infiltration (marked by blue arrows). Fenofibrate administration yielded limited amelioration of hepatic steatosis and inflammation, whereas both Resmetirom and compound **1d**, particularly at the 40 mg/kg dose, demonstrated substantially greater improvement in alleviating steatosis and reducing inflammatory cell infiltration (Figure 6A). Consistent with the aforementioned histological observations, the 40 mg/kg **1d** regimen significantly reduced plasma ALT and AST levels (*p* < 0.05). Similarly, Fenofibrate treatment also markedly ameliorated both ALT and AST levels (*p* < 0.05). In contrast, Resmetirom only marginally improved AST (*p* < 0.05) and failed to produce a statistically significant reduction in ALT (Figure 6B,C). Compared with Resmetirom, both 40 mg/kg **1d** and Fenofibrate demonstrated more com-prehensive improvements in plasma AST and ALT levels. The three compounds also exhibited distinct profiles in modulating plasma inflammatory cytokines: Fenofibrate significantly reduced IL-1β and IL-6 (*p* < 0.01), Resmetirom selectively suppressed IL-6 (*p* < 0.001), whereas both 20 and 40 mg/kg **1d** concurrently attenuated TNFα, IL-1β, and IL-6 levels (*p* < 0.001; Figure 6D–F). Notably, compared with Fenofibrate and Resmetirom, **1d** treatment produced a broader anti-inflammatory effect.

### 2.6. ***1d*** Ameliorates Hepatic Lipid Metabolism in MCD Diet-Induced MASH Mice by Modulating the AMPK-ACC-PPARα Axis

**Figure 7:**
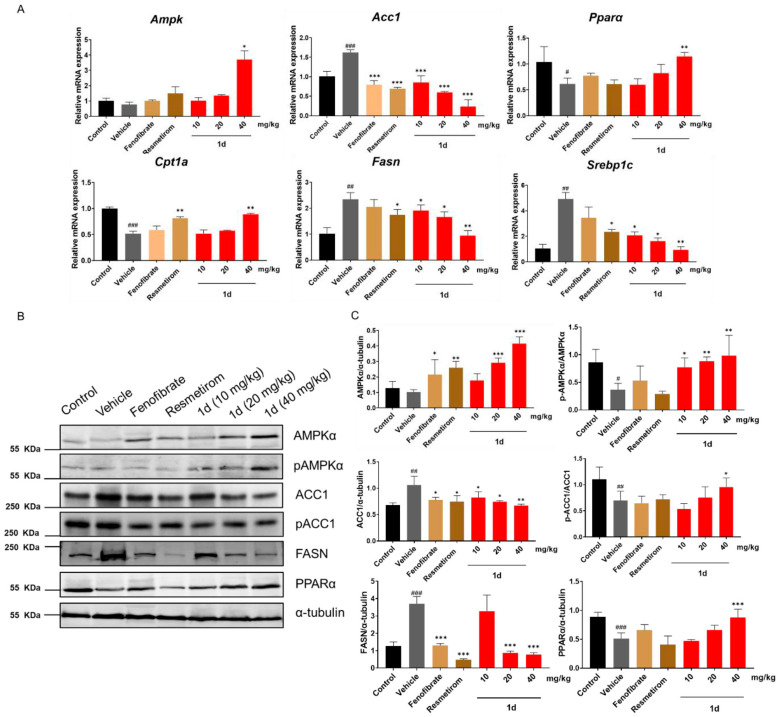
**1d** regulates transcription and expression of lipid metabolism-related genes through AMPK-ACC-PPARα axis (**A**) mRNA levels of hepatic lipid metabolism-related genes in different treatment groups. (**B**,**C**) Protein expression levels and densitometric quantification of key components in the AMPK/ACC/PPARα pathway in liver tissues. Data are presented as mean ± SD (*n* = 3). Statistical significance: ^#^ *p* < 0.05, ^##^ *p* < 0.01, ^###^ *p* < 0.001 vs. Control; * *p* < 0.05, ** *p* < 0.01, *** *p* < 0.001 vs. Vehicle. The color scheme for each group is consistent with Figure 5.

To further investigate the mechanism by which compound **1d** ameliorates hepatic lipid accumulation and metabolic dysregulation in MCD diet-induced MASH mice, we analyzed the mRNA expression changes in key genes involved in fatty acid synthesis and β-oxidation in liver tissues (Figure 7A and Figure S1). Treatment with **1d** significantly downregulated the mRNA levels of genes associated with lipid accumulation, including *Srebp1c*, *Fasn*, *Apob*, and *Acc1* (*p* < 0.05). At the 40 mg/kg dose, **1d** markedly upregulated the expression of key β-oxidation-related genes such as *Acox1*, *Cpt1a*, and *Pparα* (*p* < 0.01). Among these, *Acox1* and *Cpt1a* are established target genes of *PPARα*, whereas *Srebp1*, *Fasn*, and *Apob* are known to be indirectly regulated by PPARα.

Notably, the 40 mg/kg **1d** treatment also significantly increased the mRNA level of *Ampk* (*p* < 0.05; Figure 7A). This intriguing finding prompted us to hypothesize that the lipid-lowering effect of **1d** may involve modulation of the AMPK-ACC-PPARα axis. Given the critical role of protein phosphorylation in the AMPK/ACC pathway, we further examined the expression of key proteins in this signaling cascade (Figure 7B,C). The results demonstrated that the 40 mg/kg **1d** treatment not only enhanced AMPK expression but also increased the pAMPK/AMPK ratio (*p* < 0.01), indicating activation of AMPK. Concurrently, it reduced ACC expression while elevating the pACC/ACC ratio (*p* < 0.05). Downstream, FASN expression was significantly suppressed (*p* < 0.001), whereas PPARα expression was upregulated (*p* < 0.05).

## 3. Discussion

In previous studies, we demonstrated that the pan-PPAR agonist **1d** improves insulin resistance in T2DM mice by inhibiting PPARγ Ser273 phosphorylation in white adipose tissue and modulating the expression of genes such as adiponectin, without inducing weight gain or fluid retention. However, the mechanism by which **1d** ameliorates hepatic lipid accumulation in T2DM mice remained unclear. Multiple studies have underscored the strong association between T2DM and MASLD, with approximately 60% of T2DM patients affected by MASLD [55,56,57]. As a critical progressive stage of MASLD, MASH is closely linked to insulin resistance—a hallmark pathological feature of T2DM—which is also recognized as a driver of MASH progression [58,59]. Insulin resistance promotes hepatic lipid accumulation and suppresses lipolysis, leading to steatosis, which can further trigger oxidative stress and inflammation in the liver, ultimately progressing to fibrosis [60,61]. Consequently, the potential beneficial effects of T2DM therapeutics on MASLD/MASH have attracted widespread attention [62,63,64].

AMPK serves as a central regulator of cellular energy homeostasis, coordinating metabolic switching through phosphorylation of downstream target proteins [37]. It participates in a wide range of biological processes, including fatty acid and cholesterol synthesis, fatty acid oxidation, gluconeogenesis, and inflammation, rendering it an important therapeutic target for multiple metabolic diseases such as T2DM, obesity, and MASLD/MASH [35,65,66]. Given the pivotal role of PPARα in lipid metabolism, it has been recognized as a promising therapeutic target for MASH [67,68,69]. The rare dual capability of compound **1d** to simultaneously modulate the AMPK-ACC-PPARα axis and activate PPARα may underlie its comprehensive improvement of hepatic lipid metabolism and systemic lipid profiles.

Chronic liver inflammation is not only a hallmark of MASH but also a critical pathological mechanism driving hepatic fibrosis [70,71]. Hepatic steatosis resulting from disordered lipid metabolism triggers reactive oxygen species (ROS) accumulation, induces oxidative stress, and further promotes immune cell activation and hepatocyte injury [72,73]. Concurrently, chronic liver inflammation and hepatocellular injury stimulate the proliferation and activation of hepatic stellate cells (HSCs), which enhance the production and deposition of collagen and extracellular matrix (ECM), thereby accelerating the progression of liver fibrosis and cirrhosis [74,75]. Therefore, therapeutic strategies aimed at mitigating cellular damage and controlling inflammatory responses represent critical components of MASH management.

**Figure 8:**
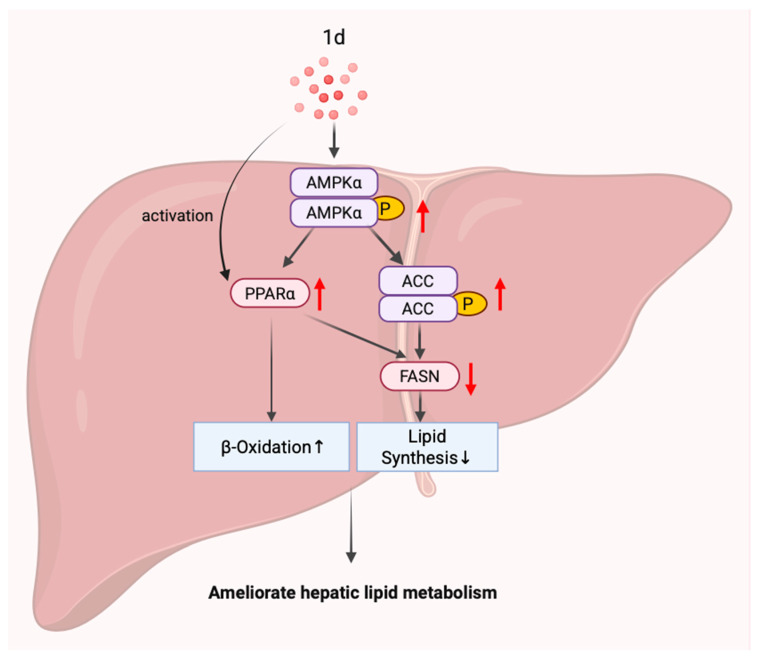
**1d** ameliorates disordered lipid metabolism through regulation of the AMPK-ACC-PPARα axis. Black arrows indicate the direction of protein regulation or changes in physiological effects, while red arrows rep-resent up- or down-regulation of protein expression levels. Black arrows indicate the direction of protein regulation or changes in physiological effects, while red arrows rep-resent up- or down-regulation of protein expression levels.

Given the complex pathophysiology of MASLD/MASH, a multi-target combination therapy approach may be necessary to achieve satisfactory clinical outcomes [76]. Our previous studies demonstrated that compound **1d**, as a pan-PPAR agonist, significantly improved insulin sensitivity and lipid metabolism in T2DM mice. The present study further reveals that compound **1d** suppresses inflammatory responses in MCD diet-induced MASH mice and ameliorates hepatic lipid metabolic disorders through modulation of the AMPK-ACC-PPARα axis. Moreover, the dual capability of **1d** to upregulate PPARα expression while simultaneously activating PPARα may synergistically enhance its promotion of fatty acid β-oxidation. However, further investigation is required to elucidate the detailed mechanisms involved (Figure 8).

## 4. Materials and Methods

### 4.1. Cell Model

The human normal hepatocyte cell line L02 and murine monocytic macrophage leukemia cell line RAW264.7 were obtained from the Cell Resource Center of Peking Union Medical College (Beijing, China). L02 cells were maintained in RPMI-1640 medium (Gibco, Grand Island, NE, USA) supplemented with 10% fetal bovine serum (Cellmax, Beijing, China) and 100 mg/L penicillin-streptomycin (Hyclone, Logan, UT, USA), and cultured at 37 °C in a humidified atmosphere containing 5% CO_2_. Similarly, RAW264.7 cells were cultured in DMEM medium (Gibco, Grand Island, NE, USA) containing 10% fetal bovine serum (Cellmax, Beijing, China) and 100 mg/L penicillin-streptomycin (Hyclone, Logan, UT, USA) under identical incubation conditions (37 °C, 5% CO_2_, humidified environment).

In the free fatty acids (FFAs)-induced cellular model of lipid accumulation, L02 hepatocytes were plated in 6-well plates at a density of 1 × 10^5^ cells per well. The cells were then co-incubated for 24 h with test compounds and a lipid mixture consisting of 0.6 mM oleic acid (OA; Solarbio Biotech, Beijing, China) and 0.3 mM palmitic acid (PA; Solarbio Biotech, Beijing, China) complexed with 5% bovine serum albumin (BSA), with 5% BSA serving as the negative control. Following treatment, intracellular lipid droplet accumulation was assessed by Oil Red O staining. For quantitative analysis, cells were lysed using CHAPS lysis buffer (Solarbio Biotech, Beijing, China), and triglyceride (TG) content was measured using a commercial TG assay kit (Nanjing Jiancheng Bioengineering Institute, Nanjing, China), with normalization to total protein concentration. Cellular ATP levels were quantified using a commercial ATP assay kit (Beyotime Biotechnology, Shanghai, China) with luminescence measurements normalized to total protein content (BCA method), while mitochondrial membrane potential (ΔΨm) was assessed by fluorescent probe staining (Solarbio Biotech, Beijing, China) followed by imaging using a laser scanning confocal microscope (Nikon A1R, Tokyo, Japan), with all experimental procedures performed in triplicate and representative images captured at 40× magnification under standardized exposure conditions.

RAW264.7 murine macrophages were seeded in 6-well plates at a density of 1 × 10^5^ cells per well and cultured for 24 h under standard conditions (37 °C, 5% CO_2_) To establish the inflammatory model, the medium was replaced with DMEM containing 1 μg/mL lipopolysaccharide (LPS) plus test compounds for an additional 24 h incubation. Nitric oxide (NO) production was quantified in culture supernatants using a commercial NO assay kit (Nanjing Jiancheng Bioengineering Institute, Nanjing, China) following the manufacturer’s protocol.

### 4.2. Cytotoxicity Assessment by MTT Assay

The in vitro cytotoxicity of test compounds was evaluated using the MTT assay. L02 cells or RAW264.7 cells were seeded in 96-well plates at a density of 1 × 10^5^ cells per well (100 µL medium per well) and allowed to adhere. After cell attachment, test compounds were added to achieve final concentrations of 5 µM, 10 µM, 20 µM, and 40 µM. The plates were incubated in a humidified atmosphere of 5% CO_2_ at 37 °C for 24 h. Following incubation, 20 µL of MTT solution (5 mg/mL) was added to each well, and the plates were further incubated for 4 h. The supernatant was carefully removed, and 100 µL of DMSO was added to each well to dissolve the formazan crystals. The absorbance was measured at 492 nm (with a reference wavelength of 630 nm) using a microplate reader (Biotek, Winooski, VT, USA), and cell viability was calculated accordingly.

### 4.3. ATP Content Quantification

Cellular ATP levels were measured using a commercial ATP assay kit (Beyotime Biotechnology, Shanghai, China) according to the manufacturer’s instructions. ATP concentrations were normalized to total protein concentration.

### 4.4. RNA Extraction and Quantitative Real-Time PCR Analysis

According to the manufacturer’s protocol (Solarbio Biotech, Beijing, China), total RNA was extracted from L02 cell line and mouse liver tissues using Triquick Reagent, with concentration determined by Nanodrop 2000 spectrophotometer (ThermoFisher Scientific, Waltham, MA, USA). Using 2 μg of total RNA, cDNA was synthesized by reverse transcription with PrimeScript™ FAST RT reagent Kit (Takara Bio, Otsu, Japan). The cDNA was then diluted 10-fold, and 2.0 μL aliquots were used as templates for quantitative PCR analysis performed on QuantStudio1 PLUS system (ThermoFisher Scientific, Waltham, MA, USA) with TB Green^®^ Premix Ex Taq™ II FAST qPCR reagents (Takara Bio, Otsu, Japan). GAPDH served as the internal reference gene for normalization, and mRNA expression levels were analyzed using specific primers listed in the Supplementary Table S1.

### 4.5. In Vivo Experimental and Tissue Collection

4–6-week-old male C57BL/6J mice (purchased from Beijing Vital River Laboratory Animal Technology Co., Ltd., Beijing, China) were housed in standard isolation cages under controlled environmental conditions (20–26 °C, 12 h light/dark cycle). After a one-week acclimation period, model group mice were switched to a methionine-choline deficient (MCD) diet and administered either solvent control, Fenofibrate (100 mg/kg), Resmetirom (10 mg/kg), or compound **1d** (10, 20, or 40 mg/kg) via oral gavage, while control group mice were fed a methionine-choline sufficient (MCS) diet. The treatment continued for 5 weeks with body weight measurements taken every three days. Fenofibrate and Resmetirom were purchased from MedChemExpress (Shanghai, China).

At the experimental endpoint, mice were fasted overnight and subsequently euthanized. Under anesthesia, blood samples were collected via the femoral artery and stored at −80 °C. Cardiac, hepatic, splenic, pulmonary, and renal tissues were rapidly frozen in liquid nitrogen and preserved at −80 °C for subsequent analysis. A portion of liver tissue was fixed in 4% paraformaldehyde for histological examination. The experimental protocol was approved by the Animal Ethics Committee of Tianjin University of Science and Technology.

### 4.6. Plasma and Hepatic Biochemical Analyses

The levels of TG, TC, LDL-C, ALT, AST in plasma or liver tissues were measured using commercial assay kits (Nanjing Jiancheng Bioengineering Institute, Nanjing, China) following the manufacturer’s protocols. Plasma levels of TNF-α, IL-1β, and IL-6 were quantified using commercial ELISA kits (Jianglai Biotechnology, Shanghai, China) in accordance with the manufacturer’s protocols.

### 4.7. Protein Isolation and Western Blot Analysis

Total proteins were extracted from mouse liver tissues using RIPA lysis buffer (Solarbio Biotech, Beijing, China), with protein concentrations determined by BCA protein assay kit (Solarbio Biotech, Beijing, China). Thirty micrograms of proteins were separated by 6% or 10% SDS-polyacrylamide gel electrophoresis (SDS-PAGE) according to target protein sizes, followed by semi-dry transfer onto pre-equilibrated polyvinylidene fluoride (PVDF) membranes (Immobilon^®^-PSQ, Merck Millipore, Darmstadt, Germany). The membranes were blocked with 1% skim milk at room temperature for 1 h and then incubated with primary antibodies at 4 °C overnight (antibody details provided in Supplementary Table S2), followed by 1 h incubation at room temperature with Donkey anti-Rabbit IgG (H + L) Highly Cross-Adsorbed Secondary Antibody, Alexa Fluor™ 680 (ThermoFisher Scientific, Waltham, MA, USA). Protein signals were detected using an infrared laser imaging system (LI-COR Biosciences, Lincoln, NE, USA), quantified by ImageJ (version 1.54) software, and normalized to α-tubulin as the internal control.

### 4.8. Hepatic Histopathological Examination

For histological analysis, liver tissues were fixed in 4% paraformaldehyde, embedded in paraffin, and sectioned at 5 μm thickness. After deparaffinization and rehydration, the sections were stained with hematoxylin and eosin (H & E) for microscopic imaging.

### 4.9. Statistical Data Analysis

The data are presented as mean ± SD, with at least three independent biological replicates per group. Differences between groups were analyzed using Statistical Product and Service Solutions (IBM, Armonk, NY, USA). One-way analysis of variance (ANOVA) was employed to determine the significance among three or more groups of replicates (* *p* < 0.05, ** *p* < 0.01, *** *p* < 0.001; ns, not significant).

## 5. Conclusions

In vitro studies demonstrated that compound **1d** effectively improved lipid metabolism and mitochondrial function, reduced lipid accumulation, and suppressed inflammatory responses. Further in vivo investigations revealed that in MCD diet-induced MASH mouse models, **1d** alleviated hepatic lipid accumulation through dual regulation of AMPK and ACC expression and phosphorylation levels, thereby modulating the AMPK-ACC-PPARα axis. The elucidation of this mechanism confirms the potential of **1d** as a promising therapeutic candidate for MASH treatment.

## Data Availability

The data presented in this study are available on request from the corresponding author due to (specify the reason for the restriction).

## Supplementary Materials

The following supporting information can be downloaded at: https://www.mdpi.com/article/10.3390/ijms262211099/s1.

## Abbreviations

The following abbreviations are used in this manuscript:

MASH	Metabolic dysfunction-associated steatohepatitis
MASLD	Metabolic dysfunction-Associated Steatotic Liver Disease
FFAs	Free Fatty Acids
MCD	Methionine-Choline Deficient
ATP	Adenosine Triphosphate
SFL	Simple Fatty Liver
HCC	Hepatocellular carcinoma
TG	Triglyceride
TC	Total Cholesterol
LDL-C	Low-Density Lipoprotein Cholesterol
THR-β	Thyroid Hormone Receptor-β
PPARs	Peroxisome proliferator-activated receptors
T2DM	Type 2 Diabetes Mellitus
LPS	Lipopolysaccharide
ROS	Reactive Oxygen Species
BSA	Bovine Serum Albumin
OA	Oleic Acid
PA	Palmitic Acid
TNFα	Tumor Necrosis Factor-α
IL-1β	Interleukin-1β
IL-6	Interleukin-6
AMPK	AMP-activated protein kinase
NO	Nitric Oxide
ACC	Acetyl-CoA carboxylase
CPT1	Carnitine palmitoyltransferase 1
FASN	Fatty Acid Synthase
CCCP	Carbonyl cyanide-chlorophenylhydrazone
ALT	Alanine Aminotransferase
AST	Aspartate Aminotransferase
