# Supplementary material for: Novel Aurone Derivative Ameliorates MASH Lipid Metabolism via the AMPK-ACC-PPARα Axis
Source: Int J Mol Sci. 2025 Nov 17;26(22):11099. doi: 10.3390/ijms262211099 (PMC12652917; doi:10.3390/ijms262211099)
Supplement: Supplementary file 1 [file ijms-26-11099-s001.zip › ijms-3982406 Supplementary Information.pdf]

Table S1. Primer sequences for qPCR

Gene		Sequences (5'→3')
SCD1(human)	Forward	TCTAGCTCCTATACCACCACCA
	Reverse	TCGTCTCCAACTTATCTCCTCC
ACLY(human)	Forward	TCGGCCAAGGCAATTTCAGAG
	Reverse	CGAGCATACTTGAACCGATTCT
ACOX1(human)	Forward	GCCATCACGCTCGGCTAATT
	Reverse	TGAGGTGGCTTGTGGTTA
SREBP1c(human)	Forward	CTTTGCCCACCCTGGTGAGT
	Reverse	GGTTCTCCTGCTTGAGTTTCTGG
FASN(human)	Forward	AAGGACCTGTCTAGGTTTGATGC
	Reverse	TGGCTTCATAGGTGACTTCCA
PPARα(human)	Forward	TCCTCGGTGACTTATCCTGT
	Reverse	CCTGTGTTCCGGTAAATGCAG
CPT1a(human)	Forward	AGCGTTCTTCGTGACGTTAG
	Reverse	CGGCCGTGTAGTAGAGATTTG
PPARγ(human)	Forward	GGGATCAGCTCCGTGGATCT
	Reverse	TGCACTTTGGTACTCTTGAAGTT
SREBP2(human)	Forward	ACAACCCATAATATCATTGAGAAACG
	Reverse	TTGTGCATCTTGGCGTCTGT
GAPDH(human)	Forward	ATGGGTGTGAACCATGAGAAG
	Reverse	GAGTCCTTCCACGATACCAAAG
Acox1(mouse)	Forward	CAGGAAGAGCAAGGAAGTGG
	Reverse	CCTTTCTGGCTGATCCCATA
Fasn(mouse)	Forward	GCTGGCATTCGTGATGGAGTCGT
	Reverse	AGGCCACCAGTGATGATGTAACTCT
Ampk(mouse)	Forward	GGTGGATTCCCAAAAGTGCT
	Reverse	AAGCAGTGCTGGGTCACAAG
Acc1(mouse)	Forward	CTCCAGGACAGCACAGATCA
	Reverse	TGACTGCCGAAACATCTCTG
PPARα(mouse)	Forward	TACTGCCGTTTTCACAAGTGC
	Reverse	AGGTCGTGTTCACAGGTAAGA
CPT1a(mouse)	Forward	TGGCATCATCACTGGTGTGTT
	Reverse	GTCTAGGGTCCGATTGATCTTTG
Apob(mouse)	Forward	GCTCAACTCAGGTTACCGTGA
	Reverse	AGGGTGTACTGGCAAGTTTGG
Srebp1c(mouse)	Forward	CGGAACCATCTTGGCAACAGT
	Reverse	CGCTTCTCAATGGCGTTGT
Gapdh(mouse)	Forward	AGGTCGGTGTGAACGGATTTG
	Reverse	TGTAGACCATGTAGTTGAGGTCA

Table S2 List of primary and secondary antibodies used in this study

Antibody	Manufacturer
Anti-AMPK alpha Rabbit pAb	Proteintech
Anti-phospho-AMPK Alpha (Thr172) Rabbit pAb	Proteintech
Anti-PPAR alpha Rabbit pAb	Proteintech
Anti-alpha Tubulin Rabbit pAb	Proteintech
Anti-FASN Rabbit pAb	Proteintech
Acetyl-CoA Carboxylase Rabbit mAb	Cell Signaling Technology
Phospho-Acetyl-CoA Carboxylase (Ser79) Rabbit mAb	Cell Signaling Technology
Donkey anti-Rabbit IgG (H+L) Highly Cross-Adsorbed Secondary Antibody, Alexa Fluor™ 680	Invitrogen

Fig.S1

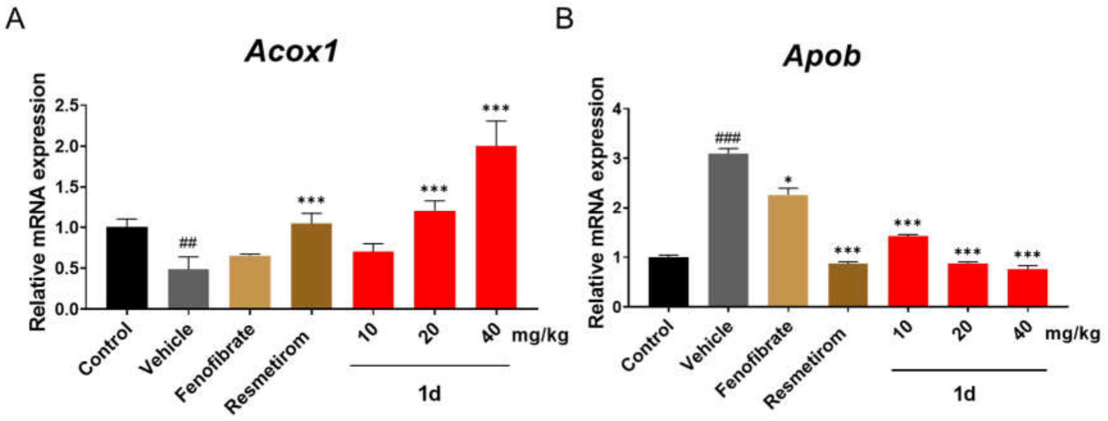

Fig. S1. Hepatic *Acox1* (A) and *Apob* (B) mRNA levels in MCD-induced MASH mice. Data are presented as mean ± SD (n = 3). Statistical significance: $^{\#}p < 0.05$, $^{\#\#}p < 0.01$, $^{\#\#\#}p < 0.001$ VS Control; $^{*}p < 0.05$, $^{**}p < 0.01$, $^{***}p < 0.001$ VS Vehicle.
